# The presence of territorial damselfish predicts choosy client species richness at cleaning stations

**DOI:** 10.1093/beheco/arac122

**Published:** 2023-02-21

**Authors:** Katie Dunkley, Kathryn E Whittey, Amy Ellison, Sarah E Perkins, Jo Cable, James E Herbert-Read

**Affiliations:** Christ’s College, University of Cambridge, Cambridge CB2 3BU, UK; Department of Zoology, University of Cambridge, Cambridge CB2 3EJ, UK; School of Biosciences, Cardiff University, Cardiff CF10 3AX, UK; School of Natural Sciences, Bangor University, Bangor, Gwynedd LL57 2UW, UK; School of Biosciences, Cardiff University, Cardiff CF10 3AX, UK; School of Biosciences, Cardiff University, Cardiff CF10 3AX, UK; Department of Zoology, University of Cambridge, Cambridge CB2 3EJ, UK; Aquatic Ecology Unit, Department of Biology, Lund University, Lund, Sweden

**Keywords:** biological market theory, cleaner fish, ecological networks, *Elacatinus evelynae*, mutualism, partner choice

## Abstract

Mutualisms are driven by partners deciding to interact with one another to gain specific services or rewards. As predicted by biological market theory, partners should be selected based on the likelihood, quality, reward level, and or services each partner can offer. Third-party species that are not directly involved in the interaction, however, may indirectly affect the occurrence and or quality of the services provided, thereby affecting which partners are selected or avoided. We investigated how different clients of the sharknose goby (*Elacatinus evelynae*) cleaner fish were distributed across cleaning stations, and asked what characteristics, relating to biological market theory, affected this distribution. Through quantifying the visitation and cleaning patterns of client fish that can choose which cleaning station(s) to visit, we found that the relative species richness of visiting clients at stations was negatively associated with the presence of disruptive territorial damselfish at the station. Our study highlights, therefore, the need to consider the indirect effects of third-party species and their interactions (e.g., agonistic interactions) when attempting to understand mutualistic interactions between species. Moreover, we highlight how cooperative interactions may be indirectly governed by external partners.

## Teaser

“Client” fish visit “cleaner” fish to have their parasites removed. Client decisions to visit one cleaner over another may not solely rely on the cleaner’s actions. Instead, clients appear to avoid cleaners where they are more likely to encounter aggressive, territorial fishes. Outcomes of species interactions, like cleaning, therefore appear to be influenced by behaviors and decisions of other species not directly involved in the cleaning interaction.

## INTRODUCTION

Mutualistic interactions often involve the beneficial exchange of goods and services between partners. Food resources, for example, can be traded for pollination, cleaning, or protection services (Hammerstein and Noë 2016). Such interactions are often characterized by a high number of different partners interacting with one another (Stanton 2003), many of which can actively choose whom to interact with (Enquist and Leimar 1993; Bshary 2001). Because individual partners differ in their likelihood or abilities to provide goods or services (Hammerstein and Noë 2016), individuals can maximize the benefits they receive by interacting with partners who provide more or higher quality services or rewards (Noë 2001). Indeed, these choices could be based on the likelihood, quality, or amount of goods or services received, otherwise known as biological market theory (Noë 2001; Bshary and Noë 2003). Such choices promote competition for the best partners: competitors should outbid others given the costs and constraints of doing so (Bshary and Noë 2003; Noe and Voelkl 2013). This partner choice therefore, is thought to play an important role in promoting the occurrence and maintenance of mutualisms (Foster and Wenseleers 2006). An individual’s choice to visit one partner over another, however, could also be affected by the presence and or behaviors of other third-party species, indirectly altering partner choice. For example, the local presence of a predator or competitor near a mutualistic partner may deter or prevent an individual entering the area and interacting with that partner (Werner and Peacor 2003). In turn, this can alter an individual’s choice to engage with one partner over another, indirectly affecting the distribution of mutualistic partners in the environment (Bronstein and Barbosa 2002). Understanding how partners are distributed across an environment and assessing the contribution of the direct (goods or services) and indirect (e.g., third-party presence and interactions) factors that may promote this differential distribution, therefore, is an important and largely unexplored extension of biological market theory.

The interactions between cleaner fish and their clients provide an ideal system for testing how direct and indirect factors affect the distribution of mutualistic partners across an environment. Cleaning involves a cleaner species, such as a fish or shrimp, removing ectoparasites, and debris from the body of another species termed a “client” (Feder 1966). Cleaners gain a source of food from the cleaning service they provide to clients, while clients benefit from parasite removal (Clague et al. 2011; Ros et al. 2020). Many cleaner species often wait at fixed territories, or cleaning stations, for clients to visit them. Clients that are able to move around the environment can hence make decisions about which cleaner(s) to interact with by visiting these cleaning stations. Cleaners can then decide whether to clean or not to clean each visiting client (Côté et al. 1998; Bshary and Noë 2003), and cleaners can differ in the cleaning service they provide (Wilson et al. 2014; Dunkley et al. 2019a). By waiting at stations, rather than roving across the reef searching for clients, cleaners appear to engage in a higher frequency of interactions with clients, and ultimately, this waiting can facilitate repeat interactions between the same cleaner and client (Soares et al. 2008a; Oates et al. 2010; Dunkley et al. 2018). By choosing which cleaning station to visit, however, client decisions to visit one station over another are ultimately governing the pool of potential clients available to the cleaners. As predicted by biological market theory, cleaners will benefit most if they interact with clients that provide more or higher quality rewards (Noë 2001). Cleaners interact with a wide number of client species (e.g., Côté et al. 1998; Grutter et al. 2003; Dunkley et al. 2019b), and as differing client species host different goods (e.g., ectoparasite diversity and abundance, Grutter 1994; Eckes et al. 2015), different client species should be expected to provide differing, as well as parallel levels, of reward quality (e.g., larger bodied species host more parasites than smaller bodied species, Poulin and Rohde 1997). For cleaners which rely on cleaning as a source of food, it would also not be advantageous to rely on the visits from one client species alone, when other client species may host similar or more beneficial rewards. For cleaners to benefit most, they should thus aim to attract a diverse and high number of clients to their station. Moreover, client species should make decisions about which cleaning stations to visit based on the likelihood, quality and quantity of the cleaning service provided, influencing the distribution of clients across local cleaning stations. To benefit most, clients should avoid visiting stations where their chances of being cleaned is low, or if the quality (duration) of the service is low, as increased cleaning durations are thought to increase the payoffs in a cleaning interaction (Gingins and Bshary 2015).

A group of client species that frequently visit cleaner fish for a cleaning service are the territorial damselfish (Pomacentridae family, Arnal and Côté 1998; Cheney and Côté 2001; Dunkley et al. 2019b). In a different context, these clients are also well known for their territorial behavior, whereby they aggressively deter some intruding species from their territories (who pose a threat to their resources) through chases, bites, and aggressive display (Bay et al. 2001). Within a reef environment, damselfish territories often overlap with cleaning stations (Arnal and Côté 1998; Whiteman et al. 2002) and thus the same client species that are visiting for a cleaning service may also be involved in these agonistic interactions with damselfishes. Such agonistic interactions may disrupt cleaning interactions, ultimately reducing the likelihood, quality, and/or quantity of the cleaning service received by visiting clients. These territorial damselfish may thus play an indirect third-party role in affecting the choices of some clients to visit one cleaning station over another (Arnal and Côté 1998; Whiteman et al. 2002).

Here, we focused on the visitation patterns of clients to cleaning stations of the Caribbean cleaner, the sharknose goby (*Elacatinus evelynae*) in relation to the likelihood and quality of service provided, and the presence of territorial third-party species (damselfishes). Cleaning stations are often visited by a number of different client species, often concurrently (Slobodkin and Fishelson 1974). Because different client species host different goods (e.g., ectoparasite diversity and abundance, Grutter 1994) and differ in their ecological overlap with damselfishes (e.g., non-algae eaters versus algae eaters), we focused on the factors that predicted the number of different client species that visited stations, that is, the species richness at a station. Some clients (residents, including damselfishes), however, are spatially restricted to which cleaning stations they can visit, since their small territories/home ranges often contain only a single cleaning station. In contrast, other client species (choosy) have larger home ranges and thus have choice options about which cleaning station(s) to visit (Bshary 2002; Bshary and Schäffer 2002). We, therefore, only considered the visitation patterns of these choosy clients across cleaning stations. To determine which factors are driving choosy clients’ decisions to visit particular cleaning stations, we identified relationships between choosy client visitation patterns, and seven factors relating to the likelihood of a client being cleaned on arrival to a station, the quality of the cleaning service provided by the cleaner(s), and the presence of territorial resident damselfish at each station. As there was a relationship between species richness and abundance at cleaning stations, whereby more choosy client species were observed at stations where there were more visits from choosy clients, we adjusted our species richness measure to account for this collinearity. We thus calculated whether the species richness of choosy clients at a station was higher or lower than expected, given the total number of client visits (by taking the residuals of the model predicting species richness as a function of client visits). We then asked whether this species richness metric changed when the likelihood, quality, and quantity of the cleaning service changed, and when there were more, or fewer, territorial damselfish visits.

## METHODS

### Quantifying client visitation and cleaning patterns at cleaning stations

Cleaning interactions between cleaners (the sharknose goby; *Elacatinus evelynae*) and their clients were observed at cleaning stations on a shallow (1–2 m water depth) fringing reef (70 m × 60 m) in Tobago (11°19.344N 060°33.484W; for more details of the site see Dunkley et al. 2019b). At this site, different client species visit coral cleaning stations occupied by cleaners (Whittey et al. 2021). Cleaners wait at their coral station for clients to visit them. Clients approach stations and can either continue swimming past or adopt a stationary pose to signal their willingness to be cleaned. Posing typically involves a client adopting a stationary head or tail-stand position with all fins flared. Cleaners can then decide to clean these visiting clients or not. A cleaning event involves the cleaner making physical contact with the body of the client to remove parasites and sometimes dead tissue, scales, or mucus (Feder 1966). While posing can increase a client’s chances of being cleaned, some client’s poses are unsuccessful, while other clients are cleaned without having to pose (Côté et al. 1998; Dunkley et al. 2018). We, therefore, quantified client visits to stations (*n* = 45, located at least 1 m apart from each other) and recorded whether each individual visit resulted in a cleaning event. To do this, 10-min observations (*n* = 208) were obtained by five different observers under snorkel over a six-week period (May—July, 2016) between the hours of 08:00 and 17:15. Choosy client visit patterns at cleaning stations did not vary with time of day (see Supplementary Figure 1). During each observation, a focal cleaner was randomly selected from the cleaning station (cleaner abundance on station ranged from one to nine individuals, mean ± 1 SD abundance = 1.24 ± 1.24) and the frequency of client visits, and cleaning events, were recorded for the focal cleaner. There is no evidence that the size of the cleaning station correlates with cleaner abundance on the station (Whittey et al. 2021). A client visit to a station was defined when an individual client was within ~20 cm of the focal cleaner, either through posing, swimming by and/or was cleaned. We used this ~20 cm swim by measure as we needed to capture all the potential clients that could have been cleaned, not just those that were actively seeking a cleaning service (i.e., through posing) and/or were cleaned. Clients vary in their tendency to pose, and cleaners also clean clients that do not pose at all (Côté et al. 1998), so our measure captures the potential pool of clients available to a cleaner. Thus, it does not bias visits to those clients that are more likely to pose and/or more likely to be cleaned. This standardized visit measure has been shown to consistently predict cleaning frequency, but not posing frequency, across 8 years of data (Dunkley et al. 2020) and represents a reasonable (and often observed) distance for a cleaner to jump onto a nearby client. For all visits, we determined whether that visit resulted in a cleaning event by the focal cleaner. Cleaning durations (seconds) were also recorded. Those clients who visited other cleaners on the station (i.e., were within ~20 cm of another non-focal cleaner), were cleaned by non-focal cleaners, or were in a large shoal, were not recorded. It was not possible to observe client visitation patterns to the entire cleaning station, or all non-focal cleaners’ cleaning behavior, due to the large three-dimensional nature of the coral head cleaning stations. For many stations, for example, it was not possible to observe cleaner behavior around multiple sides of the station at the same time. Visits and cleans used here thus represent a conservative quantification of client visit/clean frequencies to stations. Contrasting cleaner wrasse, cleaning gobies are thought to prefer consuming ectoparasites over client mucus (Soares et al. 2010) and previous studies have reported low cheating frequencies with minimal consequences on client return (Soares et al. 2008a, 2010, 2013). Thus, as cheating is unlikely in this case to influence the distributions of clients across stations in this system, data on client jolt rates (a measure used to infer a cheating event, Bshary and Grutter 2002) were not collected.

Clients were identified to a species level except for five territorial damselfish species (*Stegastes adustus*, *S. diencaeus*, *S. leucostictus*, *S. planifrons*, and *S. variabilis*) that are morphologically similar and difficult to distinguish in situ. Visit/clean events were thus combined for these five *Stegastes* spp. (as in Dunkley et al. 2019b). This did not qualitatively influence findings as we only used territorial damselfish visit frequency (grouped across multiple species, see Supplementary Table 1) in further analyses. Clients were assigned as choosy or resident using FishBase (Froese et al. 2022) with resident species defined as those whose movement is restricted to a territory (i.e., they are unlikely to move between cleaning stations). Visit (e.g., when client was within ~20 cm of focal cleaner) and clean events were also recorded for resident species as this was used to help quantify why choosy species may visit particular cleaning stations (see below). From client visitations, we identified 30 choosy client species from 37 different client species visiting cleaning stations (Supplementary Table 1). Most resident clients were territorial damselfishes. Choosy clients accounted for 43.6% of all visits to cleaning stations (860 out of 1971 visits) and 45.0% of observed cleaning events (116 out of 258 cleans).

The final durations of observation times were adjusted to account for the amount of time the cleaner was out of view (mean ± 1 SD observation time = 595 ± 11 s, minimum observation time = 552 s). Individual cleaning stations (*n* = 45) were observed between 2 and 12 times each (mean ± 1 SD number of observations per station = 4.62 ± 2.72). Differences in sampling effort between stations were accounted for in all analyses (see below) and occurred because gobies sometimes abandoned cleaning stations (gobies occupy stations for < 50 days, White et al. 2007), and because field constraints restricted our ability to sample all stations with a balanced design.

### Quantifying client richness at cleaning stations

We asked whether there was variation in the number of choosy client species (species richness) visiting each cleaning station. To do this for each station, we randomly selected two ~10 min observations and calculated the number of choosy client species visiting each station based on this subset of data, before repeating the process 1,000 times (creating *n* = 45,000 subsampled values across stations). We used the median value for each station across these simulations as our measures of richness (presented in Supplementary Figure 2). This method removes biases associated with uneven sampling effort across stations and ensures multiple combinations of observations were included in the analyses. Uneven sampling effort, for example, could increase the likelihood of finding cleaning stations with more or fewer visiting choosy client species (Vázquez et al. 2009). We checked the robustness of the species counts generated for each station using this method (see Supplementary Figure 2). Cleaning stations are often visited by the same individual client multiple times (Arnal and Côté 1998; Bshary and Schäffer 2002; Soares et al. 2008a), and in situ it was not possible to identify individual clients. Therefore, it was not possible to distinguish whether visits were from the same or multiple individuals. We therefore measured the number of client species visiting each station (species richness) rather than the abundance of each client species visiting each station.

### Why do choosy clients visit particular cleaning stations?

To identify why some stations had different visitation patterns to others, we asked whether there were relationships between visitation patterns of choosy clients to cleaning stations and the traits of the cleaning station they visited. We identified seven traits (Table 1) relating to the likelihood of receiving a cleaning service and the quality of the cleaning service. All seven traits were hypothesized to influence a choosy client’s decision to visit one cleaning station over another (see Supplementary Materials for further details on each trait). One of the seven traits related to the presence of territorial damselfish at the station (fish species considered territorial damselfish identified in Supplementary Table 1), which are predicted to disrupt cleaning interactions by chasing visiting client species (*Dunkley—personal observation*, Arnal and Côté 1998). To calculate values for five of the seven traits, we calculated multiple trait values (*n* = 1000) for each station using subsets of the original data and used median values to create a single trait value for each individual cleaning station (see Supplementary Figure 3 for distributions of each trait). This simulation method accounted for the uneven sampling effort across stations. For four traits (“Likelihood of cleaner cleaning”, “Preference for cleaning choosy client”, “Choosy client visit frequency”, and “Frequency of visits by territorial damselfish”) we calculated multiple trait values for each station using two observations per station replicated 1000 times. For “Cleaning duration” we used one observation (rather than two) per replication as there were a number of missing values for cleaning duration data (as a result of the interaction starting/ending out of view for example) and cleaning was not observed in every observation. Values for the final two traits relating to the “Likelihood of cleaner present at the station” and the “Number of cleaners on the station” were calculated using multiple presence-absence surveys (*n* = 1373, mean ± 1 SD surveys per station = 30.51 ± 9.54). These surveys recorded sharknose goby cleaner abundance at the station and were conducted at cleaning stations across the six-week study period. Correlations between each of the seven traits are presented in Supplementary Materials Table 2 and there was no evidence of multicollinearity (checked using the performance R package, Lüdecke et al. 2020, variance inflation factors all below 2.40).

**Table 1:** Traits relating to the likelihood and quality of receiving a cleaning service used to ask what factors could be driving choosy clients’ decisions to visit particular cleaning stations. Further details on how traits were calculated can be found in the Supplementary Materials. One trait value was calculated per cleaning station. Visits were recorded when a client (choosy and resident) was within ~20 cm of the focal cleaner. Clients were classified as either choosy or resident based on whether they hold resident territories on the reef. Territorial damselfish made up the majority of the resident client species (Supplementary Materials Table 1)

Trait	Measure
Choosy client visit frequency	Total number of visits to the station by choosy clients.
Likelihood of cleaner present at the station	Probability of at least one cleaner being observed on the cleaning station, measured from presence/absence data.
Number of cleaners on the station	Median number of cleaners occupying the cleaning station (from presence/absence data).
Likelihood of cleaner cleaning	Proportion of visit events that lead to a cleaning event, irrespective of client identity.
Preference for cleaning choosy client	Proportion of cleaning events that were directed at choosy clients out of all cleaning events (choosy and resident).
Cleaning duration^a^	Median cleaning duration per station (for choosy clients).
Frequency of visits by territorial damselfish	Total number of visits by territorial resident damselfish to the cleaning station.

^a^Cleaning duration data has slightly smaller sample sizes—see Supplementary Materials for further details.

There was a strong positive relationship between species richness at a station and the visit frequency of choosy clients to the station (Gaussian generalized linear model (GLM) with median species richness as the response and “Choosy client visit frequency” as the predictor variable; GLM LRT: *ß* = 0.56, *F*_1_ = 103.05, *p* < 0.001, model *R*^2^ = 0.76; see Supplementary Figure 4). Stations that were visited more frequently by choosy clients had higher species richness. We therefore used the residuals of this model as the response variable in a further Gaussian GLM asking which of the remaining six traits (Table 1) predicted the residual choosy client species richness. Here, positive residuals indicate choosy client richness at stations were higher than expected given the frequency of choosy client visits, while negative values indicate richness values were lower than expected. The full model was refined using a backwards approach and the stepAIC call from the package MASS (Venables and Ripley 2002). This approach uses Akaike information criterion to select the best model (with △AIC < 2). The significance of traits in the final model was obtained using likelihood ratio tests comparing models with and without the trait of interest. Goodness of fit was assessed using the final model’s *R*^2^ value. We also validated this result using a model averaging approach. To do this, we created an averaged model from a 95% confidence set (based on summed Akaike weights) using the model.avg call from the package MuMIn (Bartoń 2020) and identified significant traits as those whose 95% confidence intervals (calculated from model coefficients across the confidence set) did not overlap with zero (results in Supplementary Table 3). As both methods produced complimentary results, we report the results from the backwards stepwise approach. Assumptions for all models (e.g., normality of residuals, presence of influential points) were checked using the “check_model” call from the performance package (Lüdecke et al. 2020). While some studies have found a negative relationship between time of day and cleaning patterns (e.g., Sazima et al. 2000, Côté and Molloy 2003), we found no relationship between time of day, choosy client richness, and choosy client visit frequency across observations (presented in Supplementary Figure 1). It was not possible to include time of day directly in our trait model, as the richness response variable represented a median value taken across multiple observations.

Analyses were based on data from 44 out of the 45 cleaning stations: there was one station where cleaning was not observed and so this station was omitted from the analyses. In addition, as choosy clients were not observed to be cleaned at every station, there were six cleaning stations with missing values on clean duration. Rather than omitting these cases across the data, further reducing the sample size by excluding these stations, we replaced these missing values using a multiple imputation method (Nakagawa and Freckleton 2011). To do this, we used the package mice (Buuren and Groothuis-Oudshoorn 2011) to impute six missing values and repeated this process five times. This method accounts for the uncertainty around missing data by creating different combinations (*n* = 5 in this case) of plausible values for the missing data points. For each new data set we ran the model refinement process again. While cleaning duration was always retained in the final model, it remained a non-significant predictor in four of the five cases. We thus checked, and report on, clean durations significance using only the true observed data (*n* = 38 stations). This process, and the inclusion of different imputed values for clean duration, did not influence the overall result for the other six traits (all model outputs reported in Supplementary Table 3). Here, we report results from the model that had the highest *R*^2^ value (0.27, range: 0.25–0.27 across five imputed data sets, Supplementary Table 3). This imputation method therefore facilitated an analytical approach on a larger dataset for the other predictors (Nakagawa and Freckleton 2011), but its use did not qualitatively affect findings.

We assessed whether the distribution of choosy clients was spatially autocorrelated, that is were stations in close proximity of one another visited by similar species of choosy clients. To do this, we created a dissimilarity matrix quantifying the pairwise differences between which choosy client species visited each station (based on presence/absence data of each choosy client species at each station and Euclidean distance). We used a Mantel test (package ade4, Dray et al. 2007) to ask whether this client profile dissimilarity matrix correlated with a second dissimilarity matrix quantifying the pairwise distances between station locations (obtained using GPS). This test simulates a *p* value based on Monte-Carlo simulations (*n* = 999). All manuscript figures were produced with ggplot2 (Wickham 2016) and ggpubr (Kassambara 2020), and analyses were conducted in R version 4.0.2 (R Core Team 2020).

## RESULTS

**Figure 1:**
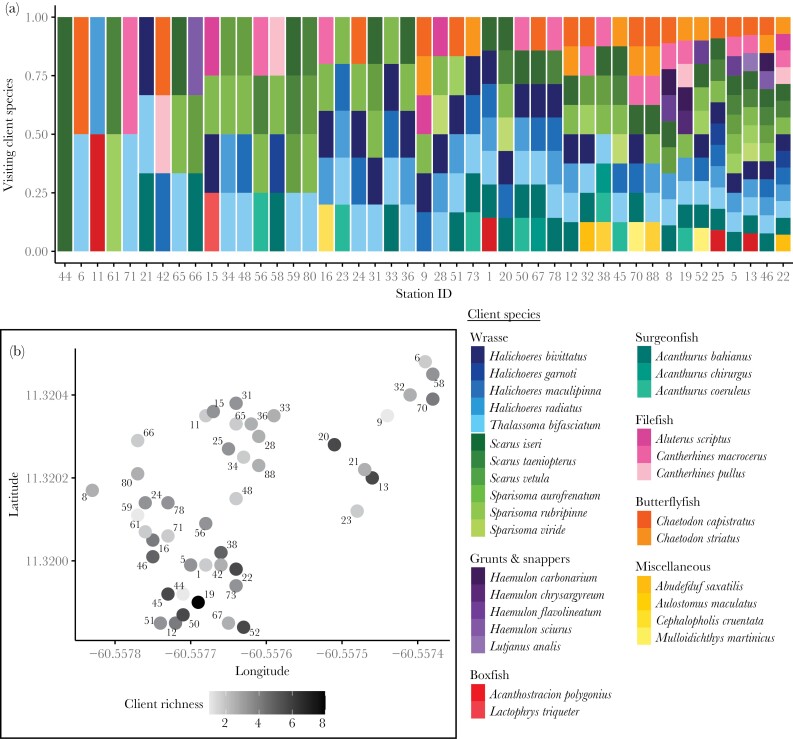
(a) Visitation patterns of 30 choosy client species to sharknose goby (*Elacatinus evelynae*) cleaning stations on Booby Reef, Man O’ War Bay Tobago. Each column represents an individual cleaning station and bars show the different choosy client species that visit each station (across all observations). Visits represent presence/absence data (e.g., client species visited station or not); therefore, each individual bar section is relative to the number of choosy client species that visited each station. Stations are ordered based on the number of choosy client species (species richness) visiting across observations. (b) Distribution of cleaning stations on Booby Reef with color showing median number of choosy client species that visited each station (richness). Median values were calculated from 1000 simulations to account for uneven sampling effort across stations: two observations per station were randomly selected, richness was quantified, and the process was repeated 1000 times. Median richness values (one per station) were used in further analysis and are shown in Supplementary Figure 2. Numbers refer to Station ID in Figure 1a.

There was variation in the number of choosy client species (species richness) that visited different cleaning stations (Figure 1). Some stations were visited by one or two different choosy species, whereas others were visited by up to 14 choosy species across the study period. As indicated by the low median value (median richness = 4, interquartile range = 3) most stations were visited by few choosy client species despite 30 identified choosy species being observed across all stations. There was no evidence that choosy client species visited stations that were spatially close to one another as the distribution of client species across stations was not spatially autocorrelated (Mantel test *r* = −0.04, *p* = 0.739, Supplementary Figure 5).

**Figure 2:**
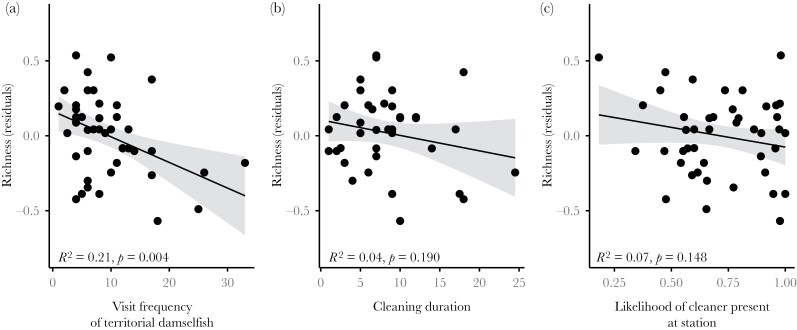
Relationships between species richness of choosy clients visiting a sharknose goby (*Elacatinus evelynae*) cleaning station and (a) the frequency of client visits to the station by territorial damselfish, (b) cleaning duration, and (c) the likelihood of a cleaner being present at the station. These three predictors were included in the final model (refined using AIC), but only the frequency of client visits to the station by territorial damselfish (a) significantly predicted species richness of choosy clients at cleaning stations (with *p* < 0.05, Supplementary Table 3). Median species richness residual values were calculated using the residuals from a GLM asking how median species richness varied as a function of choosy client visit frequency (Supplementary Figure 4). Positive values, therefore, suggest a higher choosy client richness than expected given the total frequency of client visits to the station, while negative values suggest a lower choosy client richness. Points represent adjusted values used in the model (one per station, based on median value from 1000 simulations), while lines (and shaded 95% CI) are based on predicted values from a GLM model. Individual *R*^2^ values were calculated by removing each term from the final model and subtracting the resulting *R*^2^ from the final model’s *R*^2^ (0.27).

After accounting for the strong positive relationship between choosy client species richness at a station and the number of visits to the station by choosy clients (GLM LRT: *ß* = 0.56, *F*_1_ = 103.05, *p* < 0.001, model *R*^2^ = 0.76, Supplementary Figure 4), we found that out of the six other traits, capturing the likelihood or quality of the cleaning service provided, only one predicted the distribution of choosy client species across cleaning stations. Stations had a lower species richness of choosy clients where resident territorial damselfish visitations to the cleaner were more frequent (Figure 2a, GLM LRT: *F*_1_ = 9.51, *p* = 0.004, model *R*^2^ = 0.27). Choosy client visit frequencies at each station were also lower when territorial damselfish visits to the cleaner were more frequent (Supplementary Figure 6, GLM LRT: *ß* = −0.04, *F*_1_ = 5.65, *p* = 0.022, *R*^2^ = 0.12). We found no evidence that the remaining traits predicted the distribution of choosy client species across cleaning stations (all with *p* > 0.05, Supplementary Table 3) or that the presence of other client species, which were commonly cleaned and/or frequent visitors to the station, predicted the distribution of choosy client species across cleaning stations (Supplementary Figure 7).

## DISCUSSION

Two factors predicted the uneven distribution of choosy client species across cleaning stations of the sharknose goby (*Elacatinus evelynae*) cleaner fish. First, the number of choosy client visitations to a station increased the species richness at that station. Second, species richness was higher than expected when fewer resident territorial damselfish clients visited the cleaning station. These findings highlight that the behavior of other client species appears to play an indirect role in governing observed cleaner–client interaction patterns.

Biological market theory suggests that partners should make choices of whom to interact with based on the relative value of different partners within an environment (Noë and Hammerstein 1994, 1995). Here, we develop this idea by highlighting the role that external partners and their behaviors (damselfishes) can play in indirectly altering the value of the service being provided by partners (cleaners) to others (clients). We found that the presence of territorial resident damselfish species (Pomacentridae) at a station was negatively correlated with the expected number of choosy species visiting a cleaning station (similar to findings by Arnal and Côté 1998). Damselfish are abundant on reefs and aggressively defend algae patches within their territories from intruding fish (Ceccarelli et al. 2001). This aggressive behavior can disrupt cleaning interactions by chasing away client species. For example, nearly one-third of cleaning interactions can be interrupted by one damselfish species alone (*Stegastes fuscus*, see Arnal and Côté 1998). A disrupted cleaning service will ultimately limit the rewards gained from the interaction for both choosy client and cleaner, while being chased by damselfish may be energetically costly for the client. There is conflicting evidence that damselfish territorial behavior affects other fishes’ space-use on a reef through limiting their access to certain foraging areas (Ceccarelli et al. 2001; Jones 2005; Francini-Filho et al. 2010). Here, however, territorial behavior could be indirectly promoting the uneven distribution of choosy client species visits to cleaning stations across a reef patch. Similar to a “Landscape of Fear”, which reflects a prey’s distribution in an environment as a function of its fear of predation risk (Laundre et al. 2010), certain clients appear to be choosing to visit cleaning stations where they are less likely to encounter a territorial resident damselfish. Having a resident territorial damselfish locally present at the station, therefore, is unlikely to be beneficial for the cleaner. With fewer choosy client visits, cleaners may need to rely on cleaning the potentially less rewarding resident species to gain food. Although the presence of a resident client may provide a stable and consistent daily source of food for the cleaner, these resident clients may only likely host high food supplies in the morning, with their ectoparasite loads diminishing naturally across the day (Sikkel et al. 2006) and following frequent repeat visits to cleaners (Cheney and Côté 2001). Currently, we do not have data on the distributions and abundances of damselfish territories in relation to the observed cleaning stations. Determining, however, whether cleaners show a preference for settling on coral head stations with or without resident territorial clients nearby and quantifying any differences in cleaner occupancy periods and cleaning patterns between such stations, could test for the presence of these potential trade-offs.

The disruption, or promotion, of mutualistic interactions by a non-direct partner/behavior is not uncommon across terrestrial mutualisms (e.g., Raine et al. 2002; Palmer et al. 2008; Canestrari et al. 2014), and therefore avoiding conflict with aggressive species could play a non-direct role in shaping mutualism community structure (similarly to Feeney et al. 2019). The disruption of cleaner–client interactions by territorial damselfish, however, represents a relatively unique situation: damselfish can also be involved in cleaner–client interactions as the client themselves. This poses the question whether damselfish are deterring clients visiting cleaning stations because they are attempting to monopolize access to the cleaner themselves or are simply guarding their own algal resources, reducing access to the cleaners as an indirect consequence. While algal resource guarding may play a part in deterring clients, there is also previous evidence that damselfish with cleaning stations in their territories chase other species more frequently than those damselfish whose territories do not contain cleaning stations, despite similar intrusion rates (Arnal and Côté 1998). Thus, it is not possible to rule out the hypothesis that damselfish are also monopolizing access to the cleaner(s) at the station. We found no evidence, however, that the presence of other choosy species at the cleaning station which were frequently cleaned (hence perhaps monopolizing the cleaning service), predicted the richness of other choosy clients at the cleaning station. This suggests that if damselfish are monopolizing access to the cleaner, it may not be the primary driver of why clients appear to be avoiding stations with high damselfish presence. Overall however, it was not possible here to differentiate between the likely drivers of why territorial damselfish may alter choosy clients’ visitation patterns to cleaning stations. This is because most of the observed visiting clients likely pose a threat to damselfish algal resources since they consume algae (herbivore/omnivore) or benthic invertebrates (invertivore). While algae consuming clients pose a direct threat to the algal resources of damselfish and are commonly deterred from their territories (Ceccarelli et al. 2001; Ceccarelli et al. 2005), invertivores may also pose an indirect threat through their foraging behavior. Invertivores can disturb sediments and dislodge algae resources through bioturbation (Madin et al. 2019), or consume farm-associated mysid shrimps whose waste positively benefits algal growth (Brooker et al. 2020). Indeed, several visiting invertivore clients belonged to the *Halichoeres* genus, and territorial actions of a damselfish species (*S. leucostictus*) have been shown to affect the space-use patterns of foraging *Halichoeres bivittatus* (Jones 2005). Determining how visitation patterns to cleaning stations change with the relative numbers of different choosy clients occupying different trophic levels, and when territorial residents are experimentally added or restricted from cleaning stations, is therefore needed to determine the mechanistic cause of our results. It is also important to highlight that microhabitat features of the environment may influence both the distributions of territorial damselfish on a reef and the distributions of choosy clients, although there was no evidence of spatial autocorrelation between the clients visiting stations in this study.

Contrary to predictions from biological market theory, we found no substantial evidence that the cleaners’ behavior at the station regulates choosy client visitation patterns. Stations visited by fewer choosy client species did not differ in their cleaning durations, number of cleaners, likelihood of cleaners cleaning visiting clients and cleaning preferences toward choosy versus resident clients, compared to stations that were visited by an increased species richness of choosy clients. Our findings therefore suggest that partner choice by clients may be unlikely to regulate sharknose goby cleaner–client interactions to the same extent as bluestreak wrasse (*Labroides dimidiatus*) cleaner–client interactions (Bshary 2001; Bshary and Noë 2003; Soares et al. 2008b; Adam 2010; Triki et al. 2019). In contrast to the bluestreak wrasse system (Bshary and Schäffer 2002; Soares et al. 2013; Roche et al. 2021), there is no evidence that a client’s decisions to revisit a station is based on its previous cleaning experience by Caribbean cleaning gobies, and clients do not appear to punish uncooperative cleaner behavior (Soares et al. 2008a, 2013). While the number of cleaning gobies occupying a cleaning station is usually one or two, up to nine gobies have been documented on cleaning stations within the study site. Furthermore, individual cleaning gobies occupy cleaning stations for relatively short durations (<50 days, White et al. 2007). Since individual cleaners can differ in their cleaning behavior (Dunkley et al. 2019a) and may compete with or outbid one another on the same station (Bshary and Noë 2003), re-visiting a station with increased numbers of cleaners or different cleaners, may result in inconsistent rewards to the client, even if a previous experience was positive. If clients are unable to discriminate between individual cleaners on a station, and/or dictate which cleaner cleans them, it would not be adaptive for cleaners to alter their cleaning behavior to appease clients and encourage their return. Combined with findings that cleaning service quality (duration) did not differ across stations with differing client richness; this suggests that goby cleaners may instead clean to gain rewards from the interaction without altering their service according to visiting client identity. Observed cleaning patterns may represent individual differences in the physiology, behavior or state of the cleaner goby (e.g., Dunkley et al. 2019a). Nevertheless, increased numbers of choosy client species visiting a station would benefit cleaners, as it would likely increase the quantity of food resources available (Poulin and Rohde 1997), and increase diet breadth, satisfying their energy demands (Toscano et al. 2016). Whether there is any role of cleaner preference for clients, or client choice in Caribbean cleaner–client interactions remains unclear.

Overall, our study highlights the importance of considering how the presence of other, third-party species, may influence the outcome of behavioral interactions (like mutualisms). The behaviors and actions of these species could hold indirect consequences for other interaction types. For example, agonistic interactions by territorial species could indirectly shape partner choice and the distribution of mutualistic species across the environment, which in turn, will likely link with observed interaction patterns. Adopting an experimental approach that incorporates multiple interaction types together will provide new insights into how ecological communities are structured and function through direct and indirect interactions. Recent advances in statistical analyses (e.g., multilayer network analysis) now provide a means to quantify the interconnectedness of multiple interactions. This allows us to identify how interactions feedback and influence one another (e.g., reviewed in Pilosof et al. 2017; Finn et al. 2019). Theoretical models that are used to understand and predict the decisions of cooperating species (like biological market theory) should now incorporate the behaviors and decisions of non-direct partners. This appears particularly important for cleaning goby–client interactions but could be more broadly applicable to other mutualisms and other species interactions.

## Supplementary Material

arac122_suppl_Supplementary_Material

## Notes

We thank Ryan Mohammed, Patricia Turpin (President of Environment Tobago) and Environment Research Institute Charlotteville (ERIC) for field support. We also thank Ronald Noë for his comments on a previous version of this manuscript.
