# Supplementary material for: The presence of territorial damselfish predicts choosy client species richness at cleaning stations
Source: Behav Ecol. 2023 Feb 21;34(2):269–77. doi: 10.1093/beheco/arac122 (PMC10047629; doi:10.1093/beheco/arac122)
Supplement: arac122_suppl_Supplementary_Material [file arac122_suppl_supplementary_material.pdf]

The presence of territorial damselfish predicts choosy client species richness at cleaning stations

Katie Dunkley[1,2], Kathryn E. Whittey[3], Amy Ellison[4], Sarah E. Perkins[3], Jo Cable[3], James E. Herbert-Read[1,5]

[1]Department of Zoology, University of Cambridge, Cambridge, CB2 3EJ, UK
[2]Christ's College, University of Cambridge, Cambridge, CB2 3BU, UK
[3]School of Biosciences, Cardiff University, Cardiff, CF10 3AX, UK
[4]School of Natural Sciences, Bangor University, Bangor, Gwynedd, LL57 2UW, UK
[5]Aquatic Ecology Unit, Department of Biology, Lund University, Lund, Sweden

SUPPLEMENTARY MATERIALS

Supplementary Table 1: Client species observed visiting sharknose goby (*Elacatinus evelynae*) cleaning stations. Movement and territorial information were obtained from Froese et al. (2010). *Stegastes* spp. represents five damselfish species (*Stegastes adustus, S. diencaeus, S. leucostictus, S. planifrons* and *S. variabilis*) which are morphologically similar and difficult to distinguish *in situ*.

Species	Movement	Territorial damselfish
Abudefduf saxatilis	Choosy	No
Acanthostracion polygonius	Choosy	No
Acanthurus bahianus	Choosy	No
Acanthurus chirurgus	Choosy	No
Acanthurus coeruleus	Choosy	No
Aluterus scriptus	Choosy	No
Aulostomus maculatus	Choosy	No
Cantherhines macrocerus	Choosy	No
Cantherhines pullus	Choosy	No
Canthigaster rostrata	Resident	No
Cephalopholis cruentata	Choosy	No
Chaetodon capistratus	Choosy	No
Chaetodon striatus	Choosy	No
Haemulon carbonarium	Choosy	No
Haemulon chrysargyreum	Choosy	No
Haemulon flavolineatum	Choosy	No

Haemulon sciurus	Choosy	No
Halichoeres bivittatus	Choosy	No
Halichoeres garnoti	Choosy	No
Halichoeres maculipinna	Choosy	No
Halichoeres radiatus	Choosy	No
Hypoplectrus chlorurus	Resident	No
Hypoplectrus nigricans	Resident	No
Labrisomus nuchipinnis	Resident	No
Lactophrys triqueter	Choosy	No
Lutjanus analis	Choosy	No
Microspathodon chrysurus	Resident	Yes
Mulloidichthys martinicus	Choosy	No
Scarus iseri	Choosy	No
Scarus taeniopterus	Choosy	No
Scarus vetula	Choosy	No
Sparisoma aurofrenatum	Choosy	No
Sparisoma rubripinne	Choosy	No
Sparisoma viride	Choosy	No
Stegastes partitus	Resident	Yes
Stegastes spp.	Resident	Yes
Thalassoma bifasciatum	Choosy	No

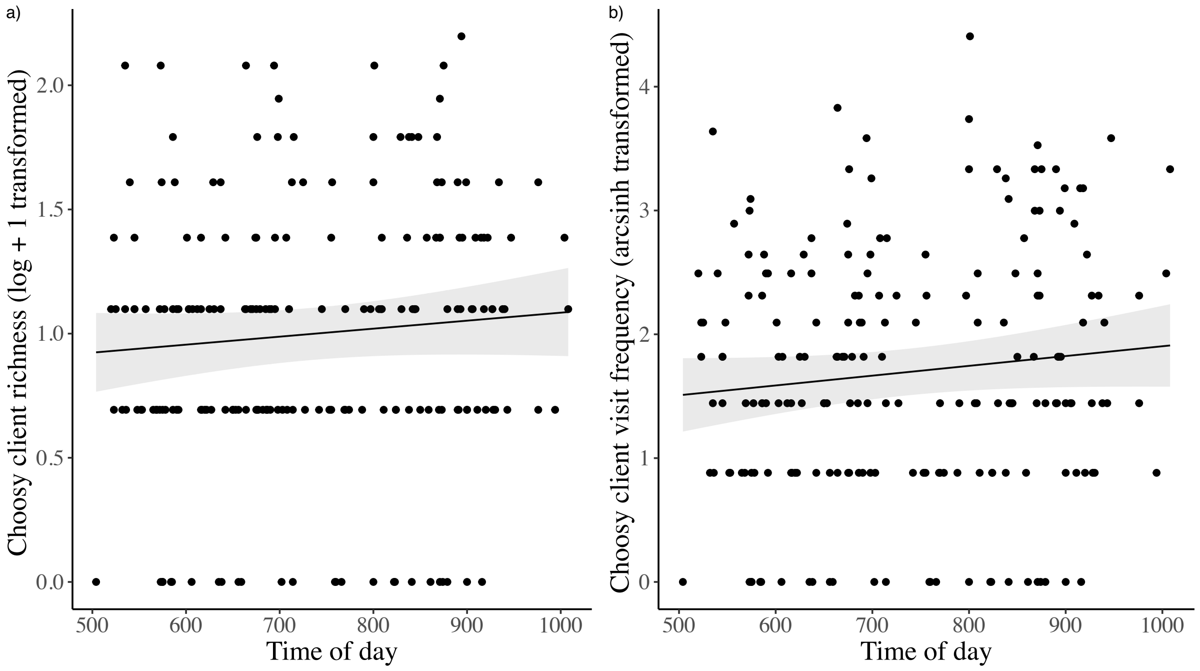

Supplementary Figure 1: Relationships between time of day and the a) the number of choosy client species (richness) visiting sharknose goby (*Elacatinus evelynae*) cleaning stations, and b) the total frequency of choosy client species visiting cleaning stations. Data are presented and analysed based on the richness values from each 10 minute observation (n = 202 observations). Time of day reflects the start time of the 10 minute observation and is included as the number of minutes since 00:00:00. We used two linear mixed models to ask if richness and choosy client visit frequency varied as a function of time of day. Station ID was included as a random term, with observation time of day (minutes since 12 am) included as a predictor. Both response variables were transformed to meet normality assumptions (richness: log + 1, visit frequency: arcsinh). Assumptions were checked with the performance package (Lüdecke et al., 2020). We found no evidence that the number of choosy client species visiting stations or the frequency of choosy client visits to stations differed across the day (choosy client richness: $X^2_1 = 1.49$, $p = 0.226$, choosy client visit frequency: $X^2_1 = 2.42$, $p = 0.120$). Richness values, adjusted for choosy client visit frequency, were included in the main analyses. Points show raw values whilst line and shaded 95% CI are based on predicted values from mixed models. Confidence intervals are calculated based on fixed effect uncertainty only.

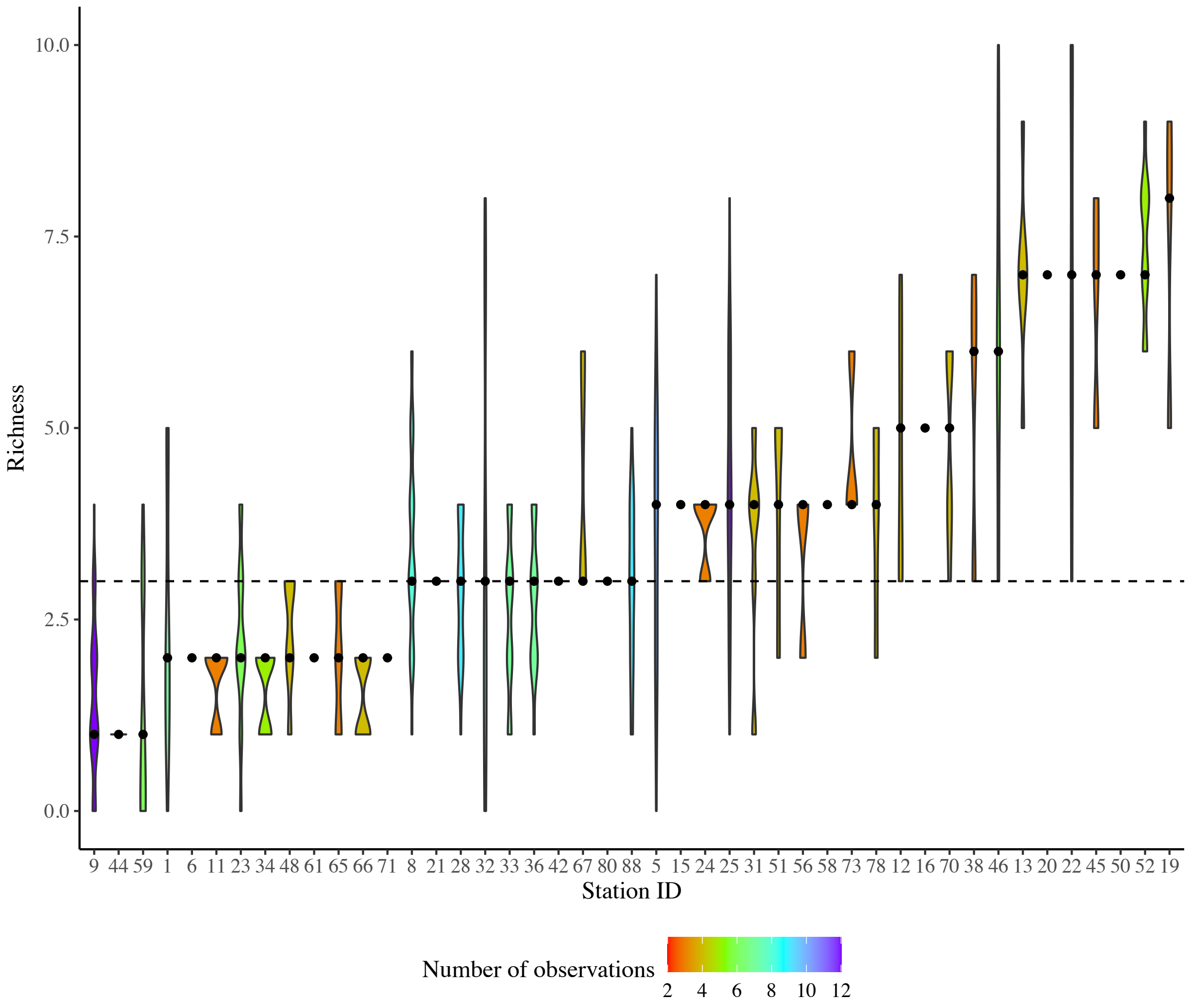

Supplementary Figure 2: Number of choosy client species (richness) visiting each sharknose goby (*Elacatinus evelynae*) cleaning station across 1,000 replications. On each replication two 10 minute observations were randomly selected for each station. Violin plot length shows the range of client species richness values, whilst shape shows how frequently these data values occurred. Stations are ordered from left to right based on their median richness (in ascending order). Individual plots are coloured based on the total number of observations per station (range: 2 - 12). Dashed line showed median richness across all stations whilst black points show the median value for each station. These median values (one per station) were used for analyses. Due to the variable sampling effort across stations it was necessary to randomly select two observations per station (repeated 1,000 times) for analysis to ensure observed numbers were not simply observed as a result of greater sampling effort. Using a Spearman's rank correlation, we found no evidence that the number of observations for each station correlated with the median richness value for each station (rho = -0.12, p = 0.449). This confirms

that our approach of taking two random observations per station (replicated 1,000 times) is appropriate for investigating patterns in species richness at cleaning stations: lower richness values are not more likely to be found at lower observation counts, whilst higher richness values are not more likely to be found at higher observation counts. Calculated richness values are therefore independent of observation count.

<u>Further details on each trait used to predict the number of choosy client species visiting each cleaning station.</u>

Unless otherwise stated, we calculated multiple trait values for each station using subsets of the original data (two observations per station) replicated 1,000 times. Median values across runs were then used as a single trait value for each individual cleaning station.

a) Likelihood of receiving cleaning service

We identified five traits relating to the likelihood of receiving a cleaning service that could influence a client's decision to visit a particular cleaning station.

When observing a cleaning station, cleaners would regularly be out of view in holes/crevices in the coral. When cleaners are out of view, visiting clients may therefore be less likely to receive a cleaning service. For each station we calculated the probability of at least one cleaner being observed on the cleaning station and used this as a measure of "*Likelihood of cleaner present at station*". To calculate this, we used cleaner presence-absence data collected in 2016 (as also used in Dunkley et al. 2020). Multiple presence-absence surveys were conducted at cleaning stations randomly throughout the day, and using these data, we calculated the proportion of times at least one *E. evelynae* cleaning goby was observed at each station.

Stations may attract more choosy clients if there are more cleaners present on the station. For example, an increased abundance of cleaners may increase the chances that a client is cleaned. For each station, using presence-absence data, we therefore calculated the median number of cleaners occupying each

station over the six-week sampling period. This provided a measure of the "*Number of cleaners on station*".

Clients can visit cleaning stations and fail to get cleaned, because cleaners choose whether to clean a client or not (Côté et al. 1998; Dunkley et al. 2018). For each station, therefore, we calculated the probability that a visit event would lead to a cleaning event, irrespective of client identity. To calculate this measure ("*Likelihood of cleaner cleaning visiting client*"), we divided the total number of cleaning events for each station by the total number of visits.

Choosy clients can select cleaner fish that give them priority access/service over resident species (Bshary and Grutter 2002). This may encourage choosy clients to visit particular stations where they are more likely to be cleaned over resident species. For each station, therefore, we calculated the likelihood that choosy clients are cleaned over resident clients ("*Preference for cleaning choosy client*"). For each station, we divided the total number of cleans towards a choosy client by the total number of cleans (for both resident and choosy clients).

Finally, clients can compete with each other for access to cleaners – clients can arrive at a cleaning station where another client may already be being cleaned, or multiple clients can arrive at a station simultaneously (Bshary and Noë 2003). This may lower the chances of the visiting client being cleaned, and therefore may choose to visit a station that is visited less frequently by clients. We therefore calculated the "Choosy client visit frequency" of each station using the total frequency of choosy client visits each station received.

b) Quality of cleaning service

Cleaners could "outbid" each other by providing a higher quality service compared to other cleaners. This outbidding could attract more clients (Bshary and Noë 2003) and increase the number of species that visit the station. We therefore identified two traits representing the relative quality of the cleaning service received at each station, to ask whether they linked with visitation patterns of choosy clients to stations.

First, an extended cleaning duration is hypothesised to increase the payoffs in a cleaning interaction, hence increasing the quality of the service (Gingins and Bshary 2015). For each station, therefore, we calculated the median cleaning duration received by choosy clients ("*Cleaning duration"*), with higher durations expected to be observed cleaning stations with higher richness. Here we used only one observation per station in subset data (replicated 1,000 times) and calculated median durations within each run. We used one observation because cleaning of choosy clients was not observed in every observation. This led to a lower number of observations that included a choosy client cleaning event (n = 63 observations where at least one choosy client cleaned). Some stations (n = 21) therefore only had one observation containing choosy client cleaning and thus it was not possible to select two observations. Cleaning of choosy clients was not observed at a further seven stations so these values were considered as missing data. Findings for cleaning duration are thus limited by the low frequency of choosy client cleaning events observed across observations (n = 116 choosy clean events across 208 station observations). Distributions of cleaning durations are shown in Supplementary Figure 3.

Areas surrounding cleaning stations are often co-inhabited by resident territorial damselfish species which aggressively defend their territory from intruding species (Arnal and Côté 1998; Whiteman et al. 2002). Whilst these territorial damselfish can visit the cleaning station(s) within their territory (be within ~ 20 cm of focal cleaner) and attempt to elicit a cleaning event, they can also disrupt other client species' cleaning interactions by chasing the client from the station (Arnal and Côté 1998). This disruption will likely reduce the pay-offs of the interaction to both cleaner and choosy client. An increased presence of a territorial damselfish at a cleaning station may thus deter choosy clients from visiting the station, reducing the number of choosy clients visiting. For each station we therefore calculated a "*Frequency of visits by territorial damselfish*" value, which represented the frequency of client visits to the station by territorial damselfish (see Supplementary Table 1 for fish species assigned as territorial damselfish).

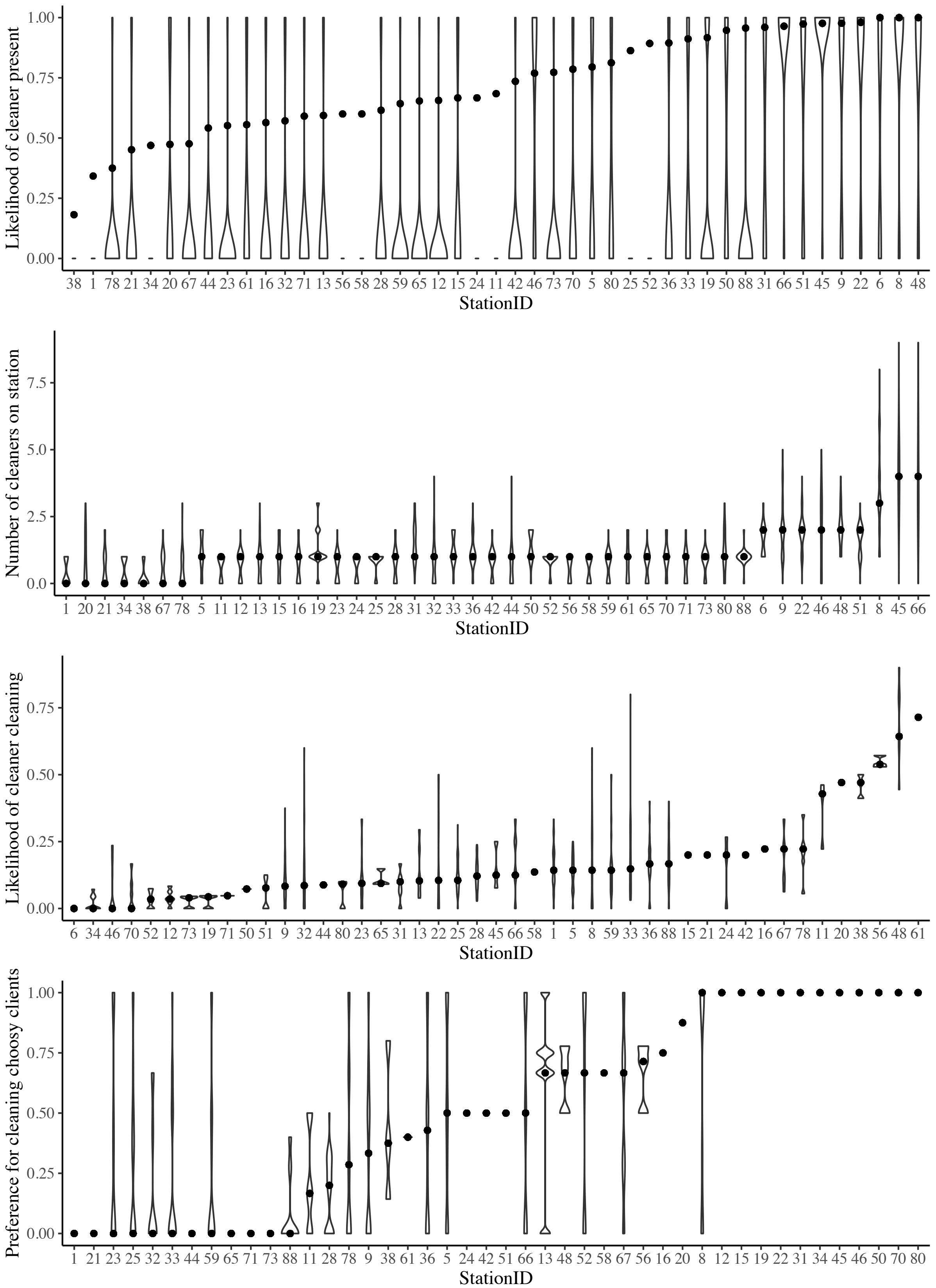
Likelihood of cleaner present
1.00
0.75
0.50
0.25
0.00
38 1 78 21 34 20 67 44 23 61 16 32 71 13 56 58 28 59 65 12 15 24 11 42 46 73 70 5 80 25 52 36 33 19 50 88 31 66 51 45 9 22 6 8 48
StationID
Number of cleaners on station
7.5
5.0
2.5
0.0
1 20 21 34 38 67 78 5 11 12 13 15 16 19 23 24 25 28 31 32 33 36 42 44 50 52 56 58 59 61 65 70 71 73 80 88 6 9 22 46 48 51 8 45 66
StationID
Likelihood of cleaner cleaning
0.75
0.50
0.25
0.00
6 34 46 70 52 12 73 19 71 50 51 9 32 44 80 23 65 31 13 22 25 28 45 66 58 1 5 8 59 33 36 88 15 21 24 42 16 67 78 11 20 38 56 48 61
StationID
Preference for cleaning choosy clients
1.00
0.75
0.50
0.25
0.00
1 21 23 25 32 33 44 59 65 71 73 88 11 28 78 9 38 61 36 5 24 42 51 66 13 48 52 58 67 56 16 20 8 12 15 19 22 31 34 45 46 50 70 80
StationID

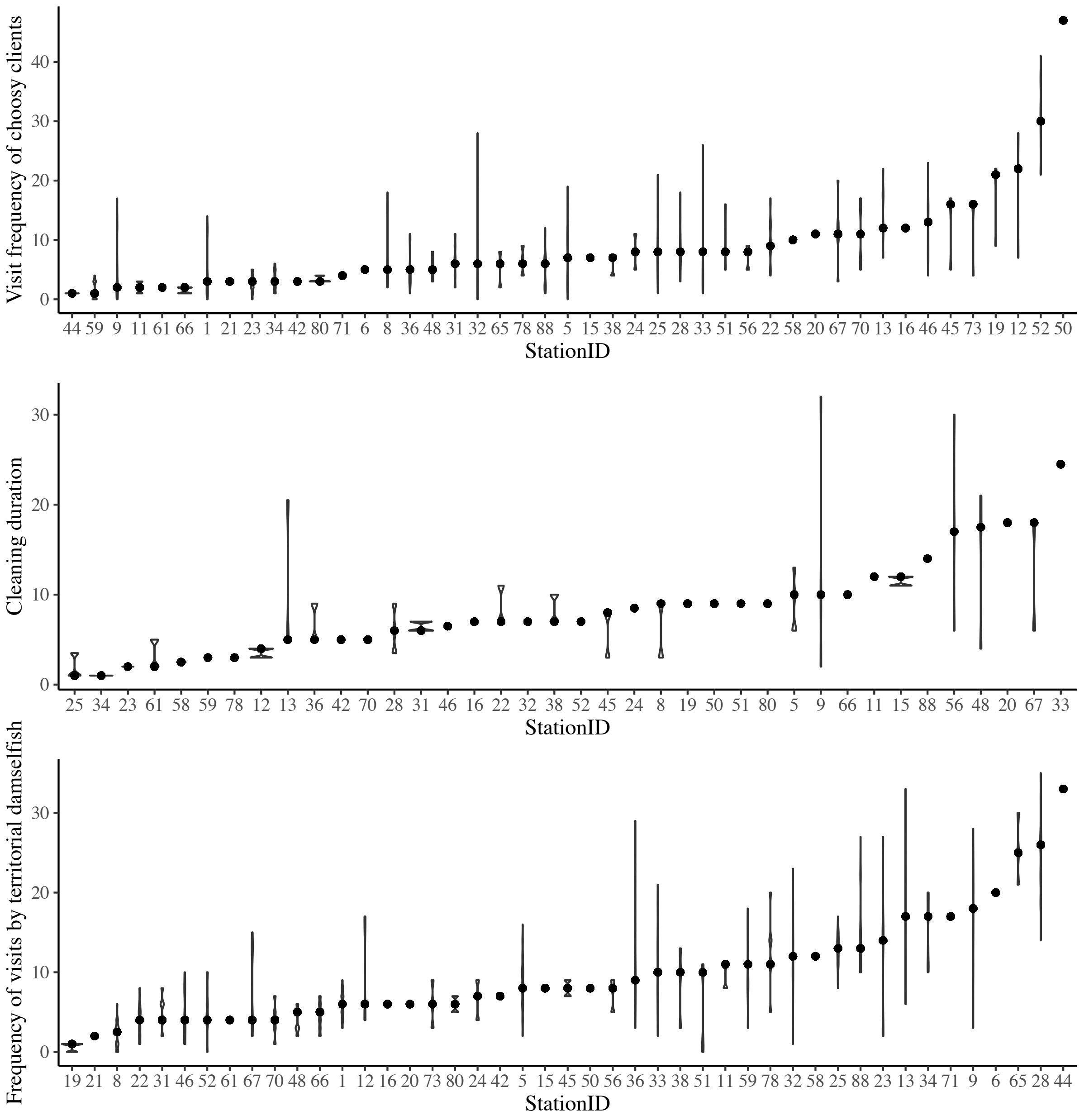

Supplementary Figure 3: Distributions of trait values used to predict choosy clients visit patterns to sharknose goby (*Elacatinus evelynae*) cleaning stations. For four traits (Likelihood of cleaner cleaning visiting client, Choosy client visit frequency, Preference for cleaning choosy client, Frequency of visits by territorial damselfish) values were calculated on two randomly selected observations (replicated 1,000 times), whilst for “Cleaning duration” median values were calculated on one randomly selected observation (replicated 1,000 times). Traits relating to the “Number of cleaners on station” and "Likelihood of cleaner present at station" were based on multiple presence-absence surveys. Violin plot length shows the range of values, whilst shape shows how frequently these data values occurred.

Stations are ordered from left to right based on their median trait value. For analyses one value (median, shown with point) was used per station.

Supplementary Table 2: Pearson's correlation coefficients between seven traits used to predict the number of choosy client species visiting sharknose goby (*Elacatinus evelynae*) cleaning stations. Correlations for all variables except cleaning duration based on n = 44 stations, whilst correlations with cleaning duration based on n = 38 stations (at six stations, choosy clients were not observed being cleaned). One missing value on "Likelihood of cleaner cleaning visiting client" was omitted from the dataset (n = 45 changed to n = 44) - cleaning was not observed at this station across the observations.

	Likelihood of cleaner present at station	Number of cleaners on station	Likelihood of cleaner cleaning visiting client	Preference for cleaning choosy client	Choosy client visit frequency	Visit frequency of territorial damselfish	Cleaning duration
Likelihood of cleaner present at station	1						
Number of cleaners on station	0.697	1					
Likelihood of cleaner cleaning visiting client	-0.26	-0.131	1				
Preference for cleaning choosy client	0.292	0.252	-0.046	1			
Choosy client visit frequency	0.249	0.013	-0.25	0.421	1		
Visit frequency of territorial damselfish	-0.228	-0.114	-0.19	-0.487	-0.257	1	
Cleaning duration	0.205	0.047	0.343	-0.001	0.028	-0.210	1

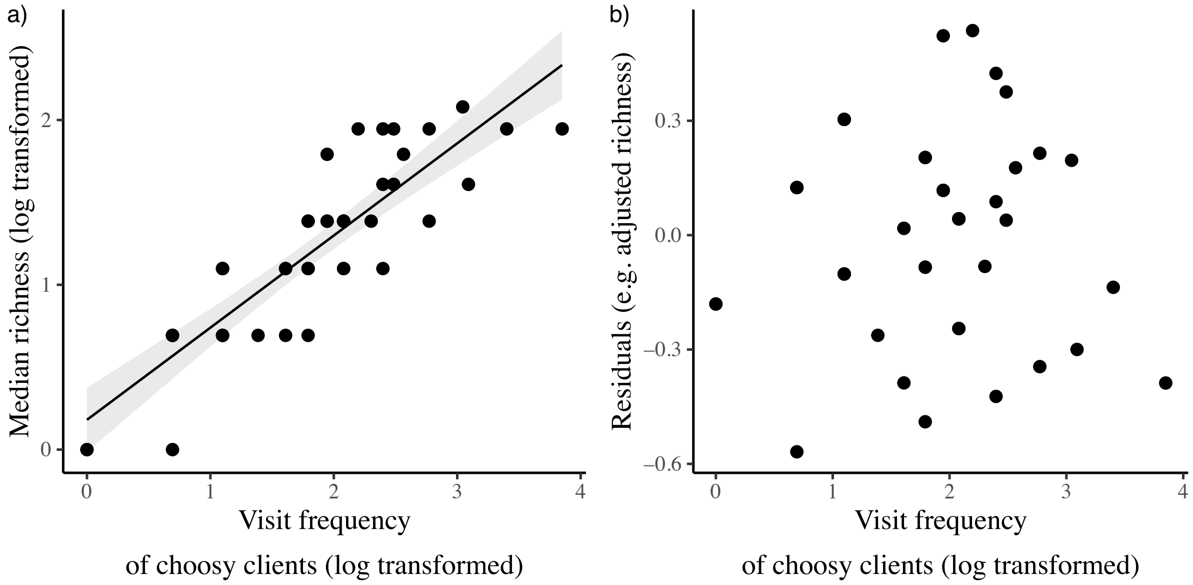

Supplementary Figure 4: a) Relationship between the median species richness of choosy clients visiting a sharknose goby (*Elacatinus evelynae*) cleaning station and the frequency of choosy client visits to the station. Median species richness values were calculated from choosy client data only, i.e. number of choosy species visiting a station, as resident clients cannot choose to visit another station. Points represent raw data points (one per station, showing median value from 1,000 simulations), whilst lines (and shaded 95% CI) are based on predicted values from a GLM model ($R^2 = 0.76$, LRT: $F_1 = 103.05$, $p < 0.001$). Median richness values were log transformed prior to modelling to improve model fit and better meet normality assumptions. Choosy client visit frequency was also included in the model as a log transformed term rather than a linear term as it better fit the data and produced a log-log linear model. We used the package bestNormalize (Peterson 2021) to identify log as the most suitable transformation for both variables. Residuals from this model (shown in b) were used in a further GLM asking which traits predict the adjusted choosy client richness at a cleaning station. b) Relationship between observed frequency of choosy client visits to the station and model residuals from (a) representing an adjusted species richness variable. b) illustrates residual fits and shows no further relationship between adjusted species richness and choosy client visit frequency.

Supplementary Table 3: Model outputs identifying which traits predict the richness of choosy clients visiting a sharknose goby (*Elacatinus evelynae*) cleaning station. Richness values represent adjusted values accounting for the frequency of choosy client visits to the station. Due to six missing values on clean duration, it was necessary to impute six values to facilitate a larger sample size. Five sets of values were imputed and a global model was run on each data set. Results represent those obtained from an AIC backwards selection approach (a) and a model averaging approach (b). The averaged model represented the top 95% of models from a model set. The model averaging approach produces values for all six traits (b), whilst only three traits were retained in the final model (a). Significant predictors of adjusted client richness within each model are highlighted in bold either using *p* values or if 95% confidence intervals (generated from non-standardised model coefficients) do not overlap with zero.

a) Model refinement with backward stepAIC

Trait	AIC	F	P	R^2	Number stations
**Data set 1**				0.26	44
Cleaning duration	5.67	3.68	0.062		
Likelihood of cleaner present at station	4.60	2.63	0.113		
Visit frequency of territorial damselfish	11.79	10.20	**0.003**		
**Data set 2**				0.27	44
Cleaning duration	5.67	**4.15**	**0.048**		
Likelihood of cleaner present at station	3.65	2.17	0.148		
Visit frequency of territorial damselfish	10.70	9.51	**0.004**		
**Data set 3**				0.26	44
Cleaning duration	5.67	3.47	0.070		
Likelihood of cleaner present at station	4.94	2.75	0.105		
Visit frequency of territorial damselfish	11.13	9.21	**0.004**		
**Data set 4**				0.25	44
Cleaning duration	5.67	2.80	0.102		
Likelihood of cleaner present at station	5.41	2.55	0.118		
Visit frequency of territorial damselfish	12.42	9.90	**0.003**		
**Data set 5**				0.25	44
Cleaning duration	5.67	2.95	0.093		

Likelihood of cleaner present at station	5.44	2.73	0.106		
Visit frequency of territorial damselfish	12.52	10.19	**0.003**		
**Data 6 - no imputed values**				0.19	38
Cleaning duration	5.22	1.79	0.190		
Likelihood of cleaner present at station	5.85	2.39	0.131		
Visit frequency of territorial damselfish	9.05	5.58	**0.024**		

b) Model averaging approach

Trait	Lower 95% CI	Upper 95% CI
**Data set 1**		
Cleaning duration	-0.0278	0.0016
Likelihood of cleaner cleaning visiting client	-0.2277	0.7974
Likelihood of cleaner present at station	-0.8049	0.1063
Number of cleaners on station	-0.1005	0.1583
Preference for cleaning choosy client	-0.0787	0.4064
Visit frequency of territorial damselfish	**-0.0278**	**-0.0022**
**Data set 2**		
Cleaning duration	**-0.0291**	**0.0000**
Likelihood of cleaner cleaning visiting client	-0.1848	0.8252
Likelihood of cleaner present at station	-0.7803	0.1334
Number of cleaners on station	-0.1008	0.1506
Preference for cleaning choosy client	-0.0808	0.3969
Visit frequency of territorial damselfish	**-0.0270**	**-0.0019**
**Data set 3**		
Cleaning duration	-0.0257	0.0020
Likelihood of cleaner cleaning visiting client	-0.2389	0.7880
Likelihood of cleaner present at station	-0.8112	0.0996
Number of cleaners on station	-0.0999	0.1608
Preference for cleaning choosy client	-0.0744	0.4079

Visit frequency of territorial damselfish	**-0.0272**	**-0.0017**
**Data set 4**		
Cleaning duration	-0.0267	0.0033
Likelihood of cleaner cleaning visiting client	-0.2453	0.7901
Likelihood of cleaner present at station	-0.8132	0.1037
Number of cleaners on station	-0.1007	0.1613
Preference for cleaning choosy client	-0.0669	0.4138
Visit frequency of territorial damselfish	**-0.0277**	**-0.0018**
**Data set 5**		
Cleaning duration	-0.0263	0.0033
Likelihood of cleaner cleaning visiting client	-0.2498	0.7822
Likelihood of cleaner present at station	-0.8165	0.0985
Number of cleaners on station	-0.1004	0.1619
Preference for cleaning choosy client	-0.0697	0.4135
Visit frequency of territorial damselfish	**-0.0279**	**-0.0020**

a)

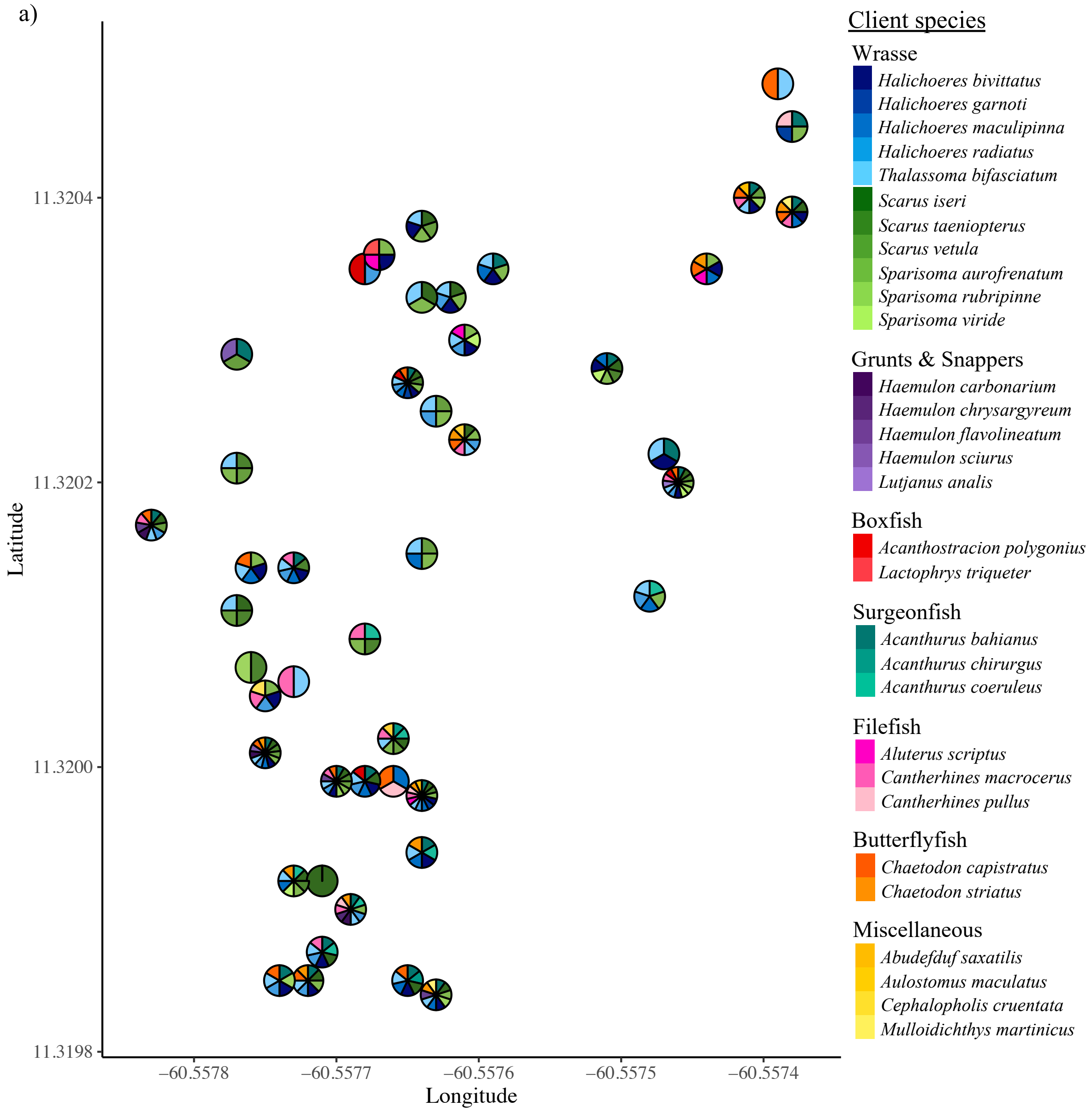
Client species
Wrasse
Halichoeres bivittatus
Halichoeres garnoti
Halichoeres maculipinna
Halichoeres radiatus
Thalassoma bifasciatum
Scarus iseri
Scarus taeniopterus
Scarus vetula
Sparisoma aurofrenatum
Sparisoma rubripinne
Sparisoma viride
Grunts & Snappers
Haemulon carbonarium
Haemulon chrysargyreum
Haemulon flavolineatum
Haemulon sciurus
Lutjanus analis
Boxfish
Acanthostracion polygonius
Lactophrys triqueter
Surgeonfish
Acanthurus bahianus
Acanthurus chirurgus
Acanthurus coeruleus
Filefish
Aluterus scriptus
Cantherhines macrocerus
Cantherhines pullus
Butterflyfish
Chaetodon capistratus
Chaetodon striatus
Miscellaneous
Abudefduf saxatilis
Aulostomus maculatus
Cephalopholis cruentata
Mulloidichthys martinicus
Latitude
11.3204
11.3202
11.3200
11.3198
Longitude
−60.5578
−60.5577
−60.5576
−60.5575
−60.5574

b)

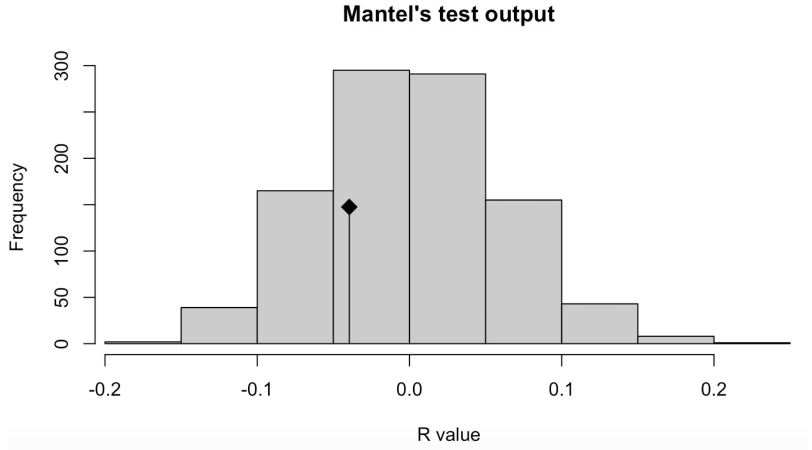
Mantel's test output
Frequency
0
50
100
200
300
-0.2
-0.1
0.0
0.1
0.2
R value

Supplementary Figure 5: a) Spatial plot showing which choosy client species visited each sharknose goby (*Elacatinus evelynae*) cleaning stations across observations. The presence/absences of each client species at stations were used to construct a dissimilarity matrix to ask whether the same choosy client species visited stations in close proximity to one another. Plot was created using package scatterpie (Yu 2021). b) Output figure from Mantel test (package ade4, Dray et al. 2007) showing observed Mantel r value (black line with square top) against the distribution of r values obtained using n = 999 Monte-Carlo simulations. There is no evidence that choosy client species simply visited stations that were in close proximity to one another.

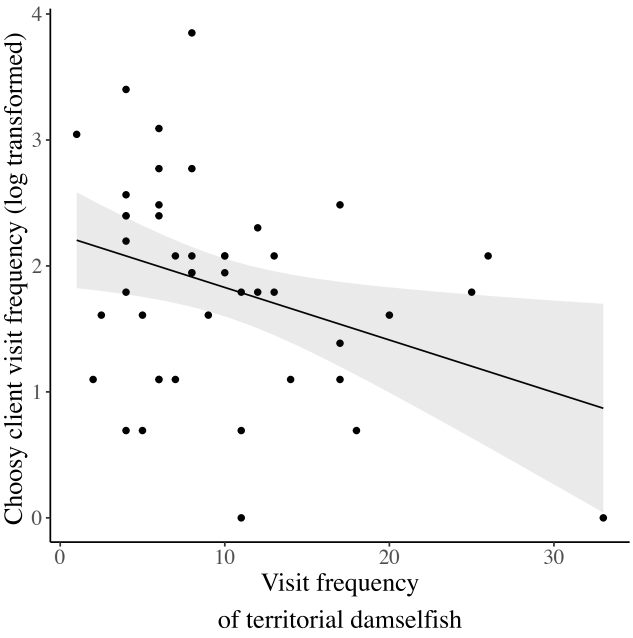

Supplementary Figure 6: Relationship between the frequency of choosy client visits to sharknose goby (*Elacatinus evelynae*) cleaning stations and the frequency of client visits to the station by territorial damselfish. Using a linear model, we found evidence that choosy client visit frequency decreased as a function of the frequency of client visits to a station by territorial damselfish ($F_1 = 5.65$, $p = 0.022$, $R^2 = 0.12$). This result is consistent with our main finding that stations which were visited more frequently by territorial damselfish, had a lower species richness of choosy clients (after accounting for visit frequency). Points represent subsampled values used in the model (one per station, based on median value from 1,000 simulations), whilst lines (and shaded 95% CI) are based on predicted values. Choosy client visit frequency was log transformed prior to analysis to meet normality assumptions. Model assumptions were checked with the performance package (Lüdecke et al., 2020).

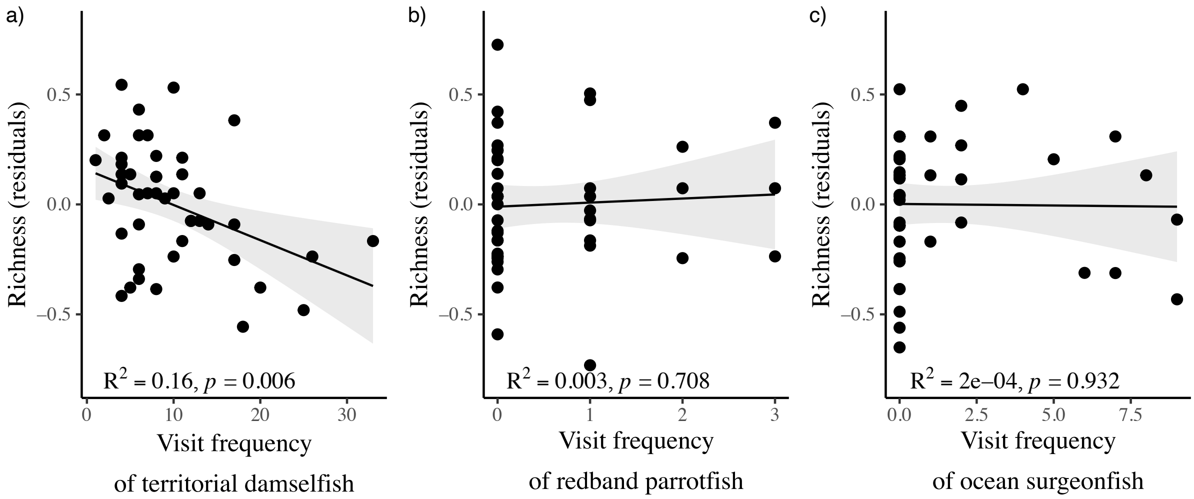

Supplementary Figure 7: Relationship between species richness of choosy clients visiting sharknose goby (*Elacatinus evelynae*) cleaning stations and the frequency of client visits to the station by a) territorial damselfish, b) redband parrotfish (*Sparisoma aurofrenatum*) and c) ocean surgeonfish (*Acanthurus bahianus*). Out of the 116 cleaning events observed towards choosy client species, the redband parrotfish was cleaned the most frequently (n = 37 events across 69 visits) while the ocean surgeonfish was the most common visitor to the stations which was also cleaned the most frequently (n = 117 visits, n = 13 cleans (third most commonly cleaned client overall)). The Queen parrotfish (*Scarus vetula*) was the second most commonly cleaned client but visited very few stations (n = 14 cleans at only 5 of the 45 stations) so was not included here. As there was no evidence for a relationship between richness values and redband parrot ($F_1 = 0.14$, $p = 0.708$), and ocean surgeonfish ($F_1 = 0.01$, $p = 0.932$), visit frequencies, this suggests that the presence of other choosy clients at cleaning stations is not altered by the presence of these two commonly cleaned species. Together this suggests that having a species that may monopolises access to cleaning services at a cleaning station does not deter other species from visiting the station. Instead, this suggests that damselfish may be deterring client species to protect their algal resources, but this requires further experimental manipulations. Median species richness residual values were calculated using the residuals from a GLM asking how median species richness varied as a function of choosy client visit frequency. Positive values suggest a higher choosy client richness than expected given the total frequency of client visits to the station, whilst negative values suggest a lower choosy client richness. For figures (b) and (c) the respective choosy client species were not included

when calculating richness values and choosy client visits to the station. Points represent adjusted values used in the model (one per station, based on median value from 1,000 simulations), whilst lines (and shaded 95% CI) are based on predicted values from a GLM model.

SUPPLEMENTARY MATERIAL REFERENCES

Arnal C, Côté IM. 1998. Interactions between cleaning gobies and territorial damselfish on coral reefs. Anim Behav. 55(6):1429–1442.

Bshary R, Grutter AS. 2002. Experimental evidence that partner choice is a driving force in the payoff distribution among cooperators or mutualists: the cleaner fish case. Ecol Lett. 5(1):130–136. doi:10.1046/j.1461-0248.2002.00295.x.

Bshary R, Noë R. 2003. The Ubiquitous Influence of Partner Choice on the Dynamics of Cleaner Fish – Client Reef Fish Interactions. Genet Cult Evol Coop.:167–184.

Côté IM, Arnal C, Reynolds JD. 1998. Variation in posing behaviour among fish species visiting cleaning stations. J Fish Biol. 53(sA):256–266. doi:10.1111/j.1095-8649.1998.tb01031.x.

Dray S, Dufour A-B, others. 2007. The ade4 package: implementing the duality diagram for ecologists. J Stat Softw. 22(4):1–20.

Dunkley K, Cable J, Perkins SE. 2018. The selective cleaning behaviour of juvenile blue-headed wrasse (*Thalassoma bifasciatum*) in the Caribbean. Behav Processes. 147:5–12. doi:10.1016/j.beproc.2017.12.005.

Dunkley K, Cable J, Perkins SE. 2020. Consistency in mutualism relies on local, rather than wider community biodiversity. Sci Rep. 10(1):21255. doi:10.1038/s41598-020-78318-x.

Froese R, Pauly D, others. 2010. FishBase. Fisheries Centre, University of British Columbia.

Gingins S, Bshary R. 2015. Pairs of cleaner fish prolong interaction duration with client reef fish by increasing service quality. Behav Ecol. 26(2):350–358. doi:10.1093/beheco/aru194.

Lüdecke D, Makowski D, Waggoner P, Patil I. 2020. performance: Assessment of Regression Models Performance. CRAN. doi:10.5281/zenodo.3952174.

Peterson RA. 2021. Finding Optimal Normalizing Transformations via bestNormalize. R J.

Whiteman EA, Côté IM, Reynolds JD. 2002. Do cleaning stations affect the distribution of territorial

reef fishes? Coral Reefs. 21(3):245–251. doi:10.1007/s00338-002-0241-x.

Yu G. 2021. scatterpie: Scatter Pie Plot. https://CRAN.R-project.org/package=scatterpie.
